# Complete mitochondrial genome of nipa palm hispid beetle *Octodonta nipae* Maulik (Coleoptera: Chrysomelidae: Cassidinae)

**DOI:** 10.1080/23802359.2021.1934172

**Published:** 2021-08-16

**Authors:** Sanqiang Yan, Baoqian Lyu, Xue Tang, Hui Lu, Jihong Tang, Rui Meng, Bo Cai, Fan Yang

**Affiliations:** a Environment and Plant Protection Institute, Chinese Academy of Tropical Agricultural Sciences, Haikou, China; b Ministry of Agriculture and Rural Affairs, Key Laboratory of Integrated Pest Management on Tropical Crops, Haikou, China; c College of Forestry, Hainan University, Haikou, China; d Post-Entry Quarantine Station for Tropical Plant, Haikou Customs, Haikou, China; e Hainan Province Engineering Research Center for Quarantine, Prevention and Control of Exotic Pests, Haikou, China

**Keywords:** *Octodonta nipae* (Maulik 1921), mitochondrial genome, phylogenetic relationship, phylogenetic analysis

## Abstract

*Octodonta nipae* (Maulik 1921) is a dangerous forestry quarantine pest, which mainly harms palms. In the present study, we determined complete mitogenome of *O*. *nipae*. This mitogenome was 15,397 bp in length (GenBank Accession no. MW802252), which contained 2 ribosomal RNA genes, 22 transfer RNAs, 13 protein-coding genes (PCGs) and one non-coding AT-rich region with the length of 883 bp. All of the 22 tRNA genes displayed a typical clover-leaf structure, with the exception of tRNA^Phe^, tRNA^Leu^, tRNA^Asn^, tRNA^Pro^ and tRNA^Thr^. Twelve PCGs were initiated by ATN codons, and NAD1 started with TTG. Ten PCGs used the typical stop codon ‘TAA’ and ‘TGA’, while three PCGs (COX2, COX3, NAD4) used the incomplete stop codons ‘TA’ or ‘T’. Phylogenetic tree demonstrated that *O*. *nipae* belongs to the family Chrysomelidae and closer to the superfamily Cassidinae.

*Octodonta nipae* (Maulik 1921), a dangerous quarantine pest for forestry originating from Malaysia (Fu et al. 2020). The adults and larvae of *O*. *nipae* gather together to feed on young stems, young shoots and heart leaves of palm plants (Yu et al. 2009). Since first discovered in 2001 in China, it has caused serious damage to nursery stock for palm plants, ecological environment, and city afforestation (Fu et al. 2020). To date, no mitogenome has been studied for this beetle.

In this paper, individuals of *O*. *nipae* were collected from Xixiu Beach Park in Haikou (110°15′36″N, 20°1′48″E), Hainan, China. The specimens were deposited at −20 °C in the herbarium of Post-Entry Quarantine Station for Tropical Plant, Haikou Customs District, PR China (Meng Rui, 541946422@qq.com), under the Accession no. IN06091001-0001-0003. Individual hind legs were used to extract DNA. The mitogenome sequence of *O*. *nipae* was generated using Illumina HiSeq X TEN Sequencing System and assembled by MitoZ software without parameters (Meng et al. 2019).

The complete circular mitogenome of *O*. *nipae* had a length of 15,397 bp (Genbank Accession no. MW802252). The nucleotide composition of *O*. *nipae* mitogenome was biased toward AT at 74.1%, and the total base composition was 40.0% A, 34.1% T, 9.3% G, and 16.6% C. The circular genome contained two ribosomal RNA genes, 22 transfer RNAs, 13 protein-coding genes (PCGs), and 1 non-coding AT-rich region with the length of 883 bp. The order and orientation of the above mitochondrial genes were typical of other leaf beetle and identical to those of the ancestral insects (Guo et al. 2017; Liu et al. 2018; Meng et al. 2019).

Twelve PCGs started with ATN, except that NAD1 initiated with TTG. 10 PCGs used the typical stop codon ‘TAA’ and ‘TAG’, while three PCGs (COX2, COX3, NAD4) used the incomplete stop codons ‘TA’ or ‘T’. All of the 22 tRNAs have a typical clover-leaf secondary structure, except for tRNA^Phe^, tRNA^Leu^, tRNA^Asn^, tRNA^Pro^, and tRNA^Thr^ whose TΨC arm formed a simple loop. All tRNAs had normal lengths, which varied from 61 to 70 bp. The 16S rRNA was 1338 bp long with an AT content of 78.6%, while the 12S rRNA was 792 bp long with an AT content of 78.0%. The non-coding region (putative control region) was 883 bp that located between 12S rRNA and tRNA^Ile^.

The concatenated datasets of the 13 PCGs from mitogenome of 18 Chrysomeloidea species from NCBI were adopted to build phylogenetic tree. *Lampyris noctiluca* and *Photinus pyralisd* that from Elateroidea were used as outgroups. The analyses were performed with Bayesian inference in Phylosuite (Ronquist et al. 2012; Zhang et al. 2020). The phylogenetic tree (Figure 1) demonstrated *O. nipae* belongs to the family Chrysomelidae and closer to the superfamily Cassidinae. In conclusion, the results of this study can provide essential and important DNA molecular data for further phylogenetic and evolutionary analysis of Chrysomeloidea.

**Figure 1.**
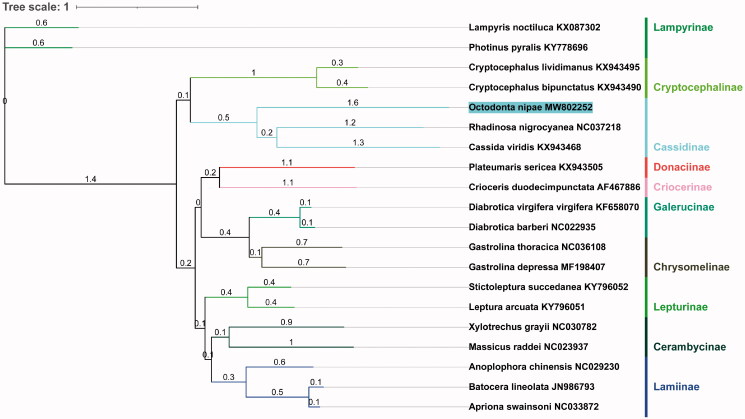
Evolutionary relationships of *O. nipae* based on mitochondrial PCGs catenated dataset. Numbers on branches are Bayesian posterior probabilities.

## Data Availability

The genome sequence data that support the findings of this study are openly available in GenBank of NCBI at (https://www.ncbi.nlm.nih.gov/) under the accession No. MW802252. The associated BioProject, Bio-Sample and SRA numbers are PRJNA723914, SAMN18837207 and SRR14306651, respectively.
